# *CDHR1*-Associated Retinal Dystrophies: Expanding the Clinical and Genetic Spectrum with a Hungarian Cohort

**DOI:** 10.3390/genes17010102

**Published:** 2026-01-19

**Authors:** Ágnes Takács, Balázs Varsányi, Mirella Barboni, Rita Vámos, Balázs Lesch, Dominik Dobos, Emília Clapp, András Végh, Ditta Zobor, Krisztina Knézy, Zoltán Zsolt Nagy, Viktória Szabó

**Affiliations:** 1 Department of Ophthalmology, Semmelweis University, Mária Str. 39, 1085 Budapest, Hungary; agnesildiko12@gmail.com (Á.T.); varsanyi.balazs@gmail.com (B.V.);; 2 Institute of Molecular and Clinical Ophthalmology, Mittlere Strasse 91, 4056 Basel, Switzerland

**Keywords:** inherited retinal dystrophy, *CDHR1* gene, c.783G>A, p.(pro261=), phenotypic variability, central and peripheral involvement

## Abstract

**Aim**: To report on the clinical and genetic spectrum of retinopathy associated with *CDHR1* variants in a Hungarian cohort. **Methods**: A retrospective cohort study was conducted at a single tertiary care referral center. The study enrolled nine patients harboring biallelic variants in the *CDHR1* gene. Detailed clinical history, multimodal imaging, electroretinography, and molecular genetics are presented. **Results**: We identified four *CDHR1* variants predicted to cause loss-of-function and five phenotypes (cone dystrophy, central areolar choroidal dystrophy, cone-rod dystrophy, rod-cone dystrophy, and late-onset macular dystrophy). The most frequent variant was the synonymous *CDHR1* c.783G>A (p.Pro261=) variant (10/18 alleles, 55.6%). A novel splice acceptor site variant, *CDHR1* c.349-1G>A, and a novel intronic variant, *CDHR1* c.1168-10A>G, were also detected. Fundus examination revealed macular atrophy with or without peripheral retinal changes. Full-field electroretinography, available in seven patients, demonstrated decreased light-adapted and extinguished dark-adapted responses in both the rod-cone dystrophy group and patients with macular involvement. OCT imaging indicated ellipsoid zone disruption with foveal sparing in two out of nine patients and severe retinal damage in rod-cone dystrophy cases. **Conclusions**: The predominant clinical manifestations of cone dystrophy, cone-rod dystrophy, and macular dystrophy in the Hungarian patient cohort showed heterogeneity, with a rod-cone dystrophy phenotype observed in five of nine cases (55.6%). The natural history of *CDHR1*-associated retinopathy typically follows a slow progression, providing a therapeutic window, which makes the disease a candidate for gene therapy.

## 1. Introduction

Inherited retinal disorders represent a colorful spectrum of various combinations of phenotypes and genotypes. To date, more than 460 retinal dystrophy genes have been described in the literature, with *CDHR1*-associated retinopathies (OMIM *609502) accounting for less than 1% of these cases [1,2,3,4].

The *CDHR1* gene (NM_033100.4), a cadherin-related family member 1, is located on chromosome 10q23.1. Its structure was first described in 2005 and was initially named PCDH21 (Protocadherin-21). It contains 17 exons, spanning 2580 base pairs, and encodes a protein of 859 amino acids [5]. The protein encoded by the *CDHR1* gene is a protocadherin, a calcium-dependent intercellular cell adhesion protein of the cadherin superfamily. It comprises three domains: six cadherin-like repeat ectodomains, a transmembrane, and an intracellular domain [6]. The *CDHR1* gene is most abundant in the nervous system and retinal tissue [7], including at the interface between the outer and inner segments of the cones and rods, and establishes structural connections between the leading edge of developing outer segment disks and the periciliary ridge of the inner segment, thereby facilitating horizontal disk elongation [5,8,9,10]. In cdhr1^−/−^ mice, disorganization of the photoreceptor outer segments, progressive photoreceptor loss, and outer retinal thinning were observed, accompanied by severe functional deficits in both dark- and light-adapted electroretinography [11]. Clinically, *CDHR1* variants are associated with cone dystrophies (COD), central areolar choroidal dystrophy (CACD), cone-rod dystrophies (CORD), rod-cone dystrophies (RCD), and late-onset macular dystrophy (LOMD) [6,12,13,14,15,16].

Based on the literature, the *CDHR1* c.783G>A, p.(pro261=) appears to be the most frequent variant [3,16]; Malechka described its occurrence in a compound heterozygous form in four out of ten cases [14], and Ba-Abbad reported one homozygous and five compound heterozygous variants from seven patients/six pedigrees [15]. There are currently over 110 variants in *CDHR1* annotated as disease-causing in the HGMD variant database [17], of which the majority (>75%) are truncating variants and <25% are missense variants [2].

In this study, we report the phenotypic and genotypic manifestations of *CDHR1* gene variants in Hungarian patients diagnosed at our clinic.

## 2. Materials and Methods

This retrospective study was conducted in accordance with the Declaration of Helsinki and with the approval of the Medical Research Council Review Board, the Health Ministry of Hungary. Informed consent was obtained from all patients.

Between April 2022 and December 2023, 530 patients with IRD at the Department of Ophthalmology at Semmelweis University underwent genetic testing. The retrospective study included data from patients with biallelic *CDHR1* gene variants.

**Clinical examination and multimodal imaging:**

In addition to standard refraction, visual acuity, and slit-lamp biomicroscopy, we conducted pedigree analysis and multimodal imaging with optical coherence tomography. The age of onset refers to when the patient first noticed the initial symptoms. The current age refers to the patient’s age at the time of the examination at our referral center.

The best-corrected visual acuity (BCVA) was determined by examining each eye separately with a Snellen chart and converting the results to logMAR. Color fundus imaging was performed by a Zeiss Clarus 700 Camera (Carl Zeiss Meditec AG, Jena, Germany) or by the conventional 35-degree Topcon Fundus Camera (Topcon, Great Britain Ltd., Berkshire, UK).

Fundus infrared and fundus autofluorescence (FAF, λ = 488 nm) imaging were obtained using a spectral domain optical coherence tomography (OCT) system (Spectralis OCT, Heidelberg Engineering Ltd., Heidelberg, Germany) to visualize cross-sectional and longitudinal structural changes.

**Electrophysiology:** The RETeval^®^ (LKC Technologies, Germantown, MD, USA) handheld portable device was used in two cases (P2 and P6) to obtain light- and dark-adapted full-field ERG recordings. In cases P1, P4, P5, P7, and P8A, full-field electroretinography was performed according to ISCEV protocols using the Roland RETIport System (Roland Consult, Brandenburg, Germany) [18].

**Genetic analysis:**

Following pretest genetic counseling, blood or oral mucosa samples were taken for genotyping. Next-generation sequencing was performed using a targeted Retinal Dystrophy Panel encompassing 314 known IRD-associated genes and 37 mitochondrial genes. Variants were categorized according to ACMG classifications, and all classified as likely pathogenic, pathogenic, or of uncertain significance (VUS) were reported [19,20,21]. During the analysis, variants classified as either likely pathogenic or pathogenic were considered confirmatory. Additional variants were considered only for genes with autosomal dominant inheritance.

## 3. Results

### 3.1. Demographics

Nine patients from seven pedigrees were ascertained (six males and three females), all harboring biallelic variants in the *CDHR1* gene. The mean age (±SD) at the time of examination was 50.5 (±14.7) years. There was no history of consanguinity in parental marriages. Three of the nine patients were related: P8A and P8B are siblings, both children of P3, whose husband was from a neighboring village. Of the nine patients, seven experienced their first symptoms in adulthood, between the ages of 25 and 50, while two had vision problems since the age of 12. The demographics and clinical information of the patients are available in Table 1. Patient follow-up data were available in eight out of nine cases. Follow-up ranged from 3 to 19 years. The average age of onset was 55.3 ± 16.7 years among patients with macular involvement and 25.16 ± 11.0 years among RCD patients.

### 3.2. Clinical Presentation and Visual Acuity

The clinical diagnosis was CACD and COD in P1 and P2, respectively. We detected late-onset macular dystrophy in P3, cone-rod dystrophy in P6, and rod-cone dystrophy in five patients (P4, P5, P7, P8A, and P8B).

All three patients diagnosed with central involvement experienced their first symptom in adulthood (between ages 44 and 74), with decreased central and color vision in two cases (P1 and P2) and photoaversion in one case (P3). Among the five patients with RCD, the first symptom occurred in adulthood in four cases (ages 25–49 years), whereas the two siblings, P8A and P8B, had symptoms since the age of 12. The first symptoms were nyctalopia in four cases (P5, P7, P8A, and P8B) and constricted visual field in three cases (P4, P5, and P8B).

Other associated eye diseases included cataract in P3, who was pseudophakic in the right eye (BCVA 0.1 LogMAR) and had nuclear cataract in her left eye (BCVA 0.3 LogMAR). Among associated systemic diseases, there was one patient (P7) who had colon cancer treated with chemotherapy at the age of 62. This patient experienced a significant deterioration in symptoms during chemotherapy and had the worst vision among the cohort of patients (hand movements BE). Data on refraction and visual acuity are shown in Table 1.

Current BCVA ranged between −0.1 LogMAR and hand movements in the total patient cohort. Mean logMAR BCVA at the first visit in the right and left eyes were 0.13 ± 0.05 and 0.17 ± 0.15, respectively, among patients with macular involvement, and 0.23 ± 0.22 and 0.15 ± 0.16, respectively, among RCD patients. The average logMAR BCVA change in the right and left eyes was −0.05 ± 0.07 and 0.05 ± 0.07, respectively, among patients with macular involvement, and 0.58 ± 0.66 in the right eye and 0.65 ± 0.77 on the left eye among RCD patients. There was a greater difference between the two phenotype groups regarding current mean logMAR BCVA, 0.1 ± 0.14 in the right eye and 0.15 ± 0.07 on the left eye among patients with macular involvement, and 0.81 ± 0.78 in the right eye and 0.8 ± 0.89 on the left eye among RCD patients.

Two patients (P1 and P2) diagnosed with CACD and COD, and P3 with LOMD, had relatively good vision on the chart (between 0.3 logMAR and −0.1 logMAR). There was no remarkable progression in the vision of patients (PI, P2) diagnosed with CACD and COD during follow-up. The P3 with LOMD had only one visit. Five patients with RCD showed a larger range of BCVA. Two patients (P7 and P8A) had a BCVA of 1.0 LogMAR or worse. All RCD patients showed notable progression in logMAR BCVA during the follow-up.

### 3.3. Fundus Appearance (Figure 1, Figure 2 and Figure 3)

Color fundus photographs were available in seven out of nine patients. We found abnormalities in the macular area in all nine cases. Among the three patients with macular involvement, two had atrophy (P1, P2), one had pigment deposition (P1), and one had only mild pigment alteration (P3). The CORD patient (P6) had multifocal macular atrophy, with some spared areas. Among the five RCD patients, four showed marked macular atrophy (P7, P8A, and P8B), two cases showed ERM (P5 and P8B), and pigment disruption was present in two cases (P4, P5).

**Figure 1:**
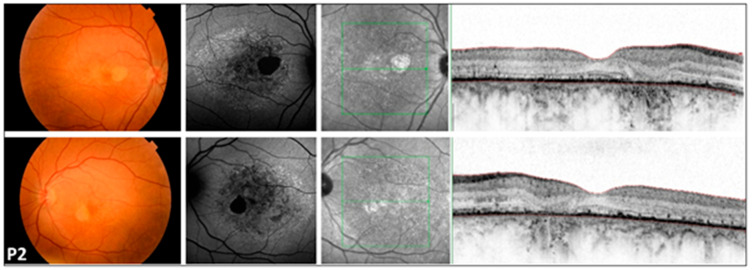
Multimodal imaging of the 46-year-old COD patient (P2) with central involvement. Images of both eyes (right in the superior, left in the bottom rows) of patient P2 with cone dystrophy, presenting a color fundus photograph, blue autofluorescent image, as well as an infrared image and a macular OCT in respective order. A symmetric bilateral small atrophic area is notable nasally from the fovea on color fundus photographs, which is consistent with a hypoautofluorescent spot surrounded by a large patchy hypoautofluorescent area on the posterior pole on FAF images. Macular OCT shows foveal sparing, severe disruption of retinal pigment epithelium, and ellipsoid zone complex. The green line indicates the locations of the scans shown in Figure 1.

**Figure 2:**
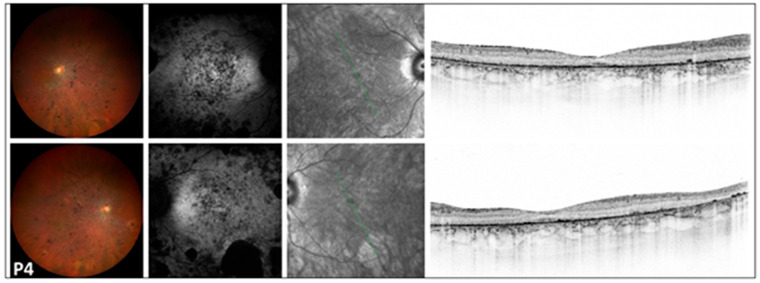
Multimodal imaging of the 54-year-old RCD patient (P4) with severe disruption of the peripheral and central retina. Images of both eyes (right in the superior, left in the bottom rows) of patient *P4* with retinitis pigmentosa presenting a color fundus photograph, blue autofluorescent image, as well as an infrared image and a macular OCT in respective order. A symmetric bilateral peripheral involvement is notable on color fundus photographs, showing significant thinning of neuroretinal layers, nummular pigmentation, pale optic disk, and attenuated vessels. FAF images show a symmetric, diffuse, patchy hypoautofluorescent area on the posterior pole, with large hypoautofluorescent spots along the inferotemporal arcade. Severe disruption of the peripheral and central retina was observed on OCT. The green line indicates the locations of the scans shown in Figure 2.

**Figure 3:**
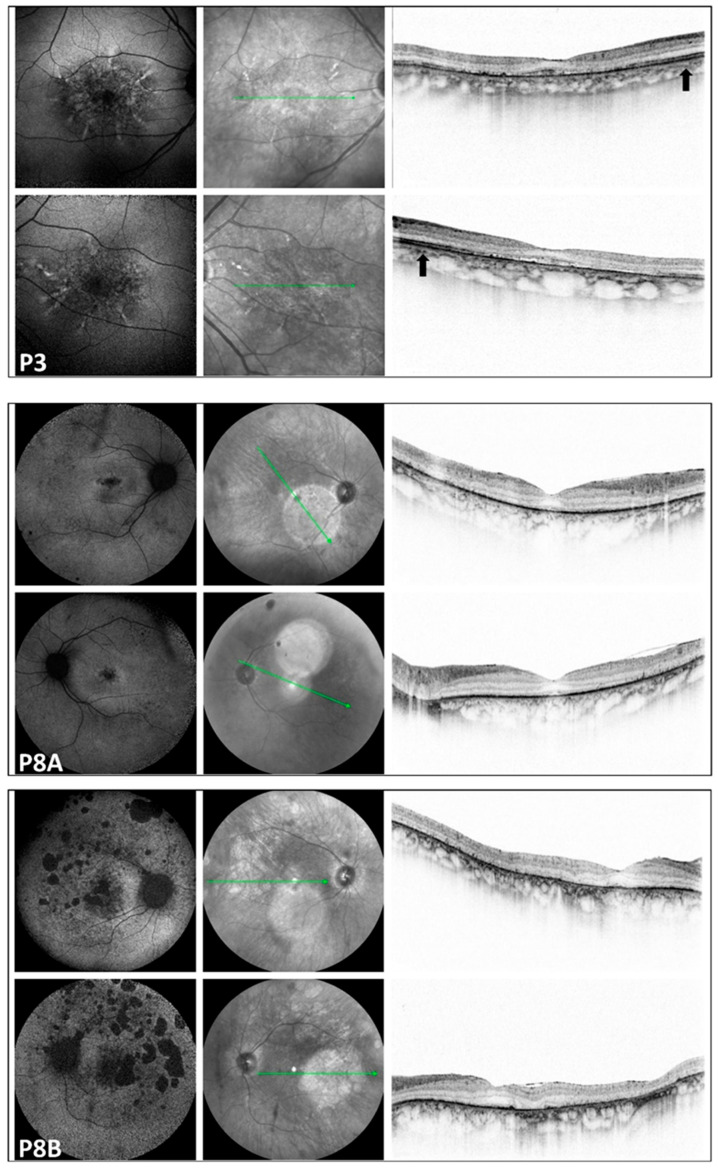
Multimodal imaging of the pedigree: P3: LOMD patient; P8A and P8B: RCD patients. Images of both eyes (right in the superior, left in the bottom rows) in respective order from the top, presenting a blue autofluorescent and an infrared image, as well as a macular OCT scan, respectively, from the right side in each row. FAF pictures of patient P3 with late-onset macular dystrophy show a diffuse pattern of dystrophy-like hypoautofluorescence, and macular OCT shows disruption of the RPE-ellipsoid zone complex. On the OCT image of P3, the arrow indicates preserved retinal layers. In the corresponding region of patients P8A and P8B, severe neuroretinal degeneration is evident. Significant disruption of the RPE-ellipsoid zone complex is observed in both the central and peripheral retina. All three patients with macular involvement exhibited a normal appearance of the optic disk and vessels. Only P3 showed some small, round pigmentation spots at the periphery; otherwise, the periphery appeared normal in the patients with macular involvement. The green line indicates the locations of the scans shown in Figure 3.

Among the five RCD patients, the following observations were made: a pale optic disk was noted in four cases (P4, P5, P7, and P8B), narrow, attenuated vessels were present in five cases (P4, P5, P7, P8A, and P8B), and bone-spicule pigmentation at the periphery was observed in three patients (P4, P5, and P7). Additionally, atrophic areas were visible at the periphery in one patient (P8A), and cobblestone degeneration with pigment deposits was noted in another patient (P8B).

### 3.4. Multimodal Imaging (Figure 1, Figure 2 and Figure 3)

FAF imaging was performed in all patients (nine out of nine). A high degree of interocular symmetry was evident in all cases. In one case (P1), a large confluent hypoautofluorescent area was present in the posterior pole, with a small central island that remained preserved. In P2, small hyperautofluorescent spots were observed alongside a large hypoautofluorescent atrophic area, which did not affect the fovea. In the case of LOMD (P3), small perifoveal hyperautofluorescent regions were identified in the posterior pole.

FAF imaging revealed a bull’s-eye phenomenon, characterized by an extensive perifoveal hypoautofluorescent ring. This finding was noted in two cases (P5 and P7). Additionally, one case (P4) exhibited scattered tiny hypoautofluorescent and hyperautofluorescent dots. Three cases (P6, P7, and P8B) showed small or large hypoautofluorescent spots, while another patient (P8A) presented a moderately sized cluster of tiny pinprick hypoautofluorescent dots in the foveal area.

In the peripheral regions of the five RCD patients, small and large patchy hypoautofluorescent spots were observed in three cases (P4, P7, and P8B). Additionally, one patient (P8A) exhibited small hypoautofluorescent dots, while another patient (P5) displayed both small hypoautofluorescent and hyperautofluorescent dots.

### 3.5. Optical Coherence Tomography (Figure 1, Figure 2 and Figure 3)

OCT scans were available for all patients (nine out of nine). Disruption of the parafoveal ellipsoid zone was observable in all cases. In the case of P7, who had the poorest visual acuity, macula OCT showed extensive atrophy in all neuroretinal layers, with significantly widened foveal depression. Besides these qualitative findings, we also analyzed the quantitative retinal thickness of the central macula, using the central subfield thickness (CST), measuring the mean retinal thickness in the central 1 mm diameter area of the macula (Table 1). Mean CST measured on OCT of the right and left eyes were 195.66 ± 16.31 microns and 217.23 ± 23.06 microns, respectively, among patients with macular involvement, and 155.2 ± 65.78 microns and 184.16 ± 50.06 microns, respectively, among RCD patients.

### 3.6. Electrophysiological Findings

Electrophysiological data were available for seven out of the nine patients (Table 1). The patient with CACD (P1) had normal dark-adapted and light-adapted responses on full-field ERG. P2 showed slightly reduced dark-adapted and residual light-adapted responses. P6 (CORD) demonstrated slightly reduced dark-adapted and severely reduced light-adapted responses. These findings align with the clinical diagnosis of CACD and CORD.

Among the five RCD patients, four showed extinguished dark-adapted responses and residual light-adapted responses (P4, P8A, and P8B), while P7 showed extinguished dark- and light-adapted responses (Table 1). These results correlate with the severe involvement of the rod system, characteristic of RCD.

### 3.7. Molecular Genetics

We identified one family with three affected members, six sporadic cases, and five phenotypes (CACD, COD, CORD, RCD, and LOMD). Four *CDHR1* variants were identified in the cohort, predicted to cause loss of function. The most frequently detected variant was the *CDHR1* c.783G>A, p.(pro261=) out of 18 alleles (10/18 alleles, 55.6%). A novel splice acceptor site variant, c.349-1G>A, and a novel intronic variant of *CDHR1*, c.1168-10A>G, were detected. Homozygous variants were encountered in five cases (P1, P5, P7, P8a, and P8B), and all other patients were compound heterozygous (Table 2). One synonymous substitution (*CDHR1* c.783G>A (p.Pro261=), rs147346345, gNOMAD AF: 0.00446), a 7bp deletion (*CDHR1* c.2522_2528del, p.(Ile841Serfs*119, rs794727197)) and two novel intronic variants (c.349-1G>A, c.1168-10A>G) were identified. Among the five homozygous patients, two (P5 and P7) had additional pathogenic variants in other genes with autosomal dominant inheritance (Table 2).

**Figure 4:**
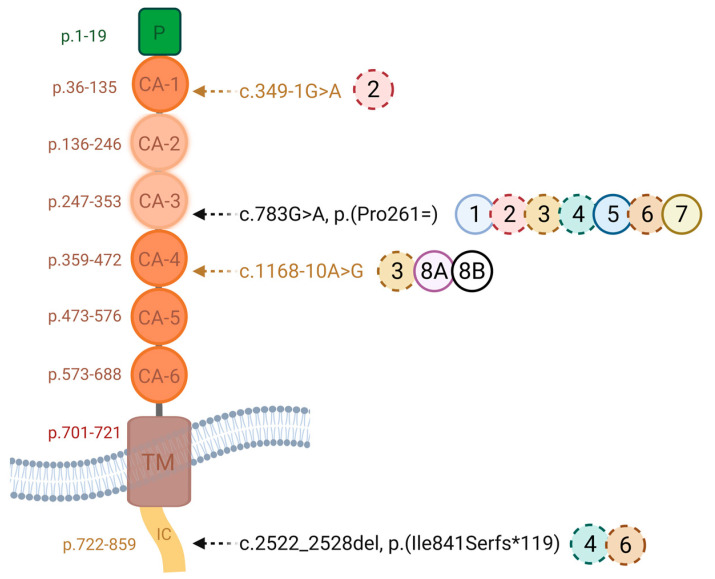
One synonymous substitution (*CDHR1* c.783G>A (p.Pro261=), rs147346345), a 7bp deletion (*CDHR1* c.2522_2528del, p.(Ile841Serfs*119, rs794727197)) and two novel intronic variants (c.349-1G>A, c.1168-10A>G) were identified. Functional minigene assay showed exon 8 skipping, which maintains the reading frame but removes 48 amino acids, disrupting the CA-2 and CA-3 cadherin domains. The solid circles indicate the homozygous cases, and dashed circles show the heterozygous cases. Each circle contains the corresponding patient number. IC: intracellular domain, TM: transmembrane domain, CA: cadherin domain (created using Biorender.com).

Figure 4 illustrates the four disease-causing variants and their locations within the cadherin domains, with the disrupted CA-2/CA-3 domains highlighted in beige.

**Figure 5:**
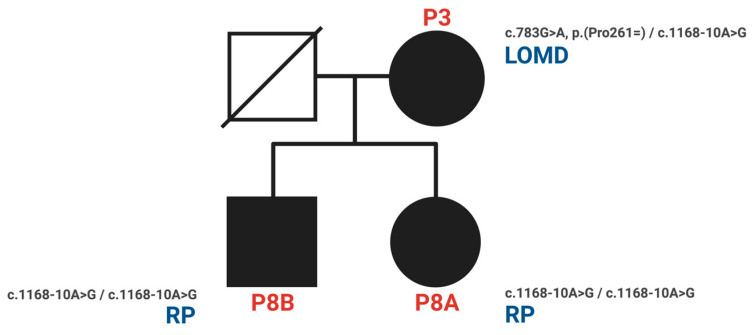
Pedigree and phenotypes of the family with the novel intronic variant *CDHR1* c.1168-10A>G (P3, P8A, P8B). (RP: retinitis pigmentosa = RCD; LOMD: late-onset macular dystrophy).

The variant *CDHR1* c.349-1G>A substitutes a nucleotide within the canonical splice acceptor site in intron 4 and is therefore likely to lead to abnormal splicing. The variant *CDHR1* c.1168-10A>G substitutes a nucleotide 10 base pairs upstream of a consensus acceptor splice site. Most in silico tools predict that this variant weakens or abolishes a splice acceptor site, whereas all predict that it creates a cryptic splice site. Sequence analysis predicts that nine nucleotides would be added to the mRNA, thus resulting in the in-frame addition of three amino acids. The variant may therefore lead to abnormal splicing and an altered protein sequence. However, these in silico predictions have not been confirmed by transcriptional studies. According to the ACMG classification, the two intronic variants (*CDHR1* c.349-1G>A, c.1168-10A>G) were classified as likely pathogenic based on the established association between the gene and the patient’s phenotype, the variant’s rarity in control populations in silico tools used to predict the substitution to alter the mRNA and protein sequence as a result of alternative splicing. The *CDHR1* c.349-1G>A variant was classified under ACMG criteria as PVS1, PM2, and PM6, while the *CDHR1* c.1168-10A>G variant met the PM2 and PP3 criteria. Segregation analysis demonstrated that homozygosity for the *CDHR1* c.1168-10A>G variant cosegregated with the corresponding phenotype in both P8A and P8B, affecting individuals of both genders. The pedigree (Figure 5) illustrates the segregation pattern of these variants; however, genotyping of the father was not possible due to his prior death.

## 4. Discussion and Conclusions

Considering the age of onset, central subfield thickness, and longitudinal changes in BCVA, our data suggest that patients with predominant macular involvement tend to present later and show slower progression than those with rod–cone dystrophy (RP). However, given the small sample size and heterogeneous follow-up intervals, robust statistical conclusions cannot be drawn. A summary of the phenotypic characteristics is provided in Table 3.

The synonymous *CDHR1* c.783G>A, p.(Pro261=) variant is well documented in *CDHR1*-associated retinal dystrophies [5,6,12,14,15,16,22]. This guanine-adenine exchange at nucleotide position 783 of the *CDHR1* gene affects the splice donor site. Although it does not change the amino acid sequence, it impacts the splice donor site at the last nucleotide of exon 8, likely leading to abnormal splicing. Functional minigene studies showed exon 8 skipping, which maintains the reading frame but removes 48 amino acids, potentially disrupting the second and third cadherin repeats (Figure 4) [6]. Consistent with previous reports, homozygous c.783G>A variants are usually linked to macula-predominant disease with relatively preserved peripheral function, often with adult-onset symptoms and near-normal or mildly reduced full-field ERG responses in early stages [6,12,14,15]. Variants affecting the canonical GU-AG nucleotides at the splice site have been reported to cause a splicing defect in numerous cases [23].

In our cohort, P1 showed a CACD phenotype consistent with the reported macula-predominant presentation in homozygous c.783G>A cases [12,14,15,24,25]. Conversely, two other homozygous c.783G>A patients (P5 and P7) exhibited apparent peripheral rod involvement, suggesting the presence of modifying factors. P5 carried an additional heterozygous likely pathogenic *RP1* variant (c.2416G>T, p.(Glu806*)), which could contribute to a more typical retinitis pigmentosa-like phenotype due to RP1’s role in outer segment disk organization [26]. Deficient expression of *RP1*, in both autosomal dominant and recessive forms, may result in the typical symptoms of retinitis pigmentosa, such as peripheral bone spicule-like pigmentation, attenuated vessels, and nyctalopia. P7, the most severely affected patient, also possessed a heterozygous *PROM1* splice-site variant (c.1301+2T>C) and experienced rapid functional decline during chemotherapy, both potentially impacting disease severity and progression [27].

Compound heterozygous c.783G>A variants have been linked to more severe disease, including peripheral degeneration [12]. In our group, two unrelated patients with compound heterozygosity (P4 and P6) carried the same *CDHR1* variant pair, including the frameshift c.2522_2528del, p.(Ile841Serfs*119) (Table 2), supporting the idea that biallelic loss-of-function mutations may lead to wider retinal involvement. The role of the additional *RGR* c.196A>C, p.(Ser66Arg) variant (VUS) remains unclear [28].

We also identified two previously unreported intronic/splice-region variants. P2 (COD phenotype; Figure 1) carried c.783G>A in trans with the novel canonical splice acceptor variant c.349-1G>A, which is expected to disrupt splicing. P3 (LOMD phenotype) carried the novel intronic variant c.1168-10A>G, predicted in silico to weaken the native acceptor site and create a cryptic splice site with an in-frame insertion of three amino acids; notably, her offspring (P8A, P8B) were homozygous for c.1168-10A>G and exhibited an RCD phenotype (Figure 3). This intrafamilial variability underscores the substantial phenotypic heterogeneity of *CDHR1*-associated retinopathy and supports the need for transcriptional studies to confirm splicing consequences and clarify genotype–phenotype relationships for these novel variants.

Overall, the high frequency of the c.783G>A allele in our cohort reinforces prior observations that this variant represents a recurrent disease-associated allele across macular phenotypes; meanwhile, peripheral degeneration can also occur, potentially driven by the position of truncating variants within cadherin domains and/or additional genetic and environmental modifiers and epigenetic factors [7,12,14,15,16,22,29,30,31,32]. Larger longitudinal cohorts and functional assays will be essential for refining prognostic counseling and better defining therapeutic windows in *CDHR1*-associated retinopathies.

## Figures and Tables

**Table 1:** Demographics and clinical data of the patients.

P No.	Age at Dg	Age	G	Family History	Refraction (SE)		BCVA LogMAR at 1st Visit		BCVA LogMAR (Current)		CST (um)		Full-Field ERG	Genotype	Phenotype
					OD	OS	OD	OS	OD	OS	OD	OS			
**P1**	Y50	Y58	M	Negative	−5.555	−5.25	0.1	0.0	0.0	0.1	211	241	normal DA and LA responses	Homozygous c.783G>A, p.(Pro261=)	**CACD**
**P2**	Y42	Y46	M	Negative	−0.5	−0.25	0.2	0.2	0.2	0.2	189	195	slightly reduced DA, residual LA responses	c.783G>A, p.(Pro261=) c.349-1G>A	**COD**
**P3**	Y74	Y76	F	Negative	−1.5	+0.375	0.1	0.3	NA	NA	187	215	NA	c.783G>A, p.(Pro261=) c.1168-10A>G	**LOMD**
**P4**	Y35	Y54	F	Negative	−0.875	−1.625	0.0	0.0	0.2	0.7	143	154	extinguished DA, residual LA responses	c.783G>A, p.(Pro261=) c.2522_2528del, p.(Ile841Serfs*119)	**RCD**
**P5**	Y25	Y29	M	Negative	−7.25	−6.125	0.4	0.1	0.7	0.1	181	227	NA	Homozygous c.783G>A, p.(Pro261=)	**RCD**
**P6**	Y30	Y46	M	Negative	0	−0.25	0.4	0.0	0.4	0.0	NA	184	slightly reduced DA, severely reduced LA responses	c.783G>A, p.(Pro261=) c.2522_2528del, p.(Ile841Serfs*119)	**CORD**
**P7**	Y37	Y73	M	Negative	−0.75	+1.75	0.5	0.3	HM	HM	47	105	extinguished DA and LA responses	Homozygous c.783G>A, p.(Pro261=)	**RCD**
**P8A**	Y12	Y47	F	Daughter of P3	−0.25	−0.125	0.1	0.4	1.0	1.4	190	192	extinguished DA, residual LA responses	Homozygous c.1168-10A>G	**RCD**
**P8B**	Y12	Y49	M	Son of P3	−0.625	+0.125	0.0	0.1	0.3	0.3	215	243	extinguished DA, residual LA responses	Homozygous c.1168-10A>G	**RCD**

P: patient, Dg: diagnosis, G: gender, DA: dark-adapted (ERGs), LA: light-adapted (ERGs).

**Table 2:** Genetic results for the Hungarian cohort presenting biallelic *CDHR1* variants, protein changes, ACMG classification, coding effects, and additional variants. (P: pathogenic, LP: likely pathogenic, AA: amino acid).

Patient No.	Allele 1				Allele 2				Additional Variants
	DNA Variant	Protein Change	ACMG	Coding Effect	DNA Variant	Protein Change	ACMG	Coding Effect	
**P1**	*CDHR1*_c.783G>A	p.(Pro261=)	**P**	Aberrant splicing	*CDHR1*_c.783G>A	p.(Pro261=)	**P**	Aberrant splicing	None
**P2**	*CDHR1*_c.783G>A	p.(Pro261=)	**P**	Aberrant splicing	*CDHR1*_c.349-1G>A	Splice acceptor variant	**LP**	Aberrant splicing (intronic)	None
**P3**	*CDHR1*_c.783G>A	p.(Pro261=)	**P**	Aberrant splicing	*CDHR1*_c.1168-10A>G	Intronic variant	**LP**	In frame addition of 3 AAs	None
**P4**	*CDHR1*_c.783G>A	p.(Pro261=)	**P**	Aberrant splicing	*CDHR1*_c.2522_2528del	p.(Ile841Serfs*119)	**P**	Frameshift variant	*RGR*_c.196A>C, p.(Ser66Arg) Heterozygous, VUS
**P5**	*CDHR1*_c.783G>A	p.(Pro261=)	**P**	Aberrant splicing	*CDHR1*_c.783G>A	p.(Pro261=)	**P**	Aberrant splicing	*RP1*_c.2416G>T, p.(Glu806*) Heterozygous, Likely Pathogen
**P6**	*CDHR1*_c.783G>A	p.(Pro261=)	**P**	Aberrant splicing	*CDHR1*_c.2522_2528del	p.(Ile841Serfs*119)	**P**	Frameshift variant	*RGR*_c.196A>C, p.(Ser66Arg) Heterozygous, VUS
**P7**	*CDHR1*_c.783G>A	p.(Pro261=)	**P**	Aberrant splicing	*CDHR1*_c.783G>A	p.(Pro261=)	**P**	Aberrant splicing	*PROM1* c.1301+2T>C Heterozygous, Pathogen
**P8A**	*CDHR1*_c.1168-10A>G	Intronic variant	**LP**	In frame addition of 3 AA’s	*CDHR1*_c.1168-10A>G	Intronic variant	**LP**	In frame addition of 3 AA’s	None
**P8B**	*CDHR1*_c.1168-10A>G	Intronic variant	**LP**	In frame addition of 3 AA’s	*CDHR1*_c.1168-10A>G	Intronic variant	**LP**	In frame addition of 3 AA’s	None

**Table 3:** Characteristics of phenotypes associated with *CDHR1* variants: central areolar choroidal dystrophy (CACD), cone dystrophies (COD), cone-rod dystrophies (CORD), rod-cone dystrophies (RCD, RP), and late-onset macular dystrophy (LOMD).

Phenotypes	CACD	COD	CORD	LOMD	RCD
**Onset**	45–50 ys	35–40 ys	30–40 ys	late (over 60 ys)	childhood
**Macular** **involvement**	severe atrophy in the macula with preserved islands	severe atrophy in the macula	severe atrophy in the macula	mild atrophy in the macula	patchy atrophy in the macula
**Peripheral** **involvement**	None	None	Yes	None	Yes

## Data Availability

The data presented in this study are available on request from the corresponding author. The data are not publicly available due to privacy or ethical restrictions.

## Abbreviations

ACMG	American College of Medical Genetics and Genomics
AMD	age-related macular degeneration
BCVA	best corrected visual acuity
CACD	central areolar choroidal dystrophy
CRT	central retinal thickness
COD	cone dystrophy
CORD	cone-rod dystrophy
EZ	ellipsoid zone
IRD	inherited retinal disease
LOMD	late-onset macular dystrophy
LP	likely pathogenic
P	pathogenic
NGS	next-generation sequencing
OCT	optical coherence tomography
OD	right eye
OS	left eye
RCD	rod-cone dystrophy
RP	retinitis pigmentosa
RPE	retinal pigment epithelium
SD	standard deviation
VUS	variant of uncertain significance
